# CDK19 as a Potential HPV-Independent Biomarker for Recurrent Disease in HNSCC

**DOI:** 10.3390/ijms21155508

**Published:** 2020-07-31

**Authors:** Finn-Ole Paulsen, Christian Idel, Julika Ribbat-Idel, Patrick Kuppler, Luise Klapper, Dirk Rades, Karl-Ludwig Bruchhage, Barbara Wollenberg, Johannes Brägelmann, Sven Perner, Anne Offermann

**Affiliations:** 1 Institute of Pathology, University of Luebeck and University Hospital Schleswig-Holstein, Campus Luebeck, Ratzeburger Allee 160, 23538 Luebeck, Germany; finn-ole.paulsen@uksh.de (F.-O.P.); Julika.Ribbat-Idel@uksh.de (J.R.-I.); patrick.kuppler@student.uni-luebeck.de (P.K.); luise.klapper@student.uni-luebeck.de (L.K.); 2 Department of Otorhinolaryngology, University of Luebeck, Ratzeburger Allee 160, 23538 Luebeck, Germany; Christian.Idel@uksh.de (C.I.); Karl-Ludwig.Bruchhage@uksh.de (K.-L.B.); 3 Department of Radiation Oncology, University of Luebeck, Ratzeburger Allee 160, 23538 Luebeck, Germany; dirk.rades@uksh.de; 4 Department of Otorhinolaryngology, University Hospital MRI, Technical University, Ismaningerstraße 22, 81675 Munich, Germany; barbara.wollenberg@tum.de; 5 Molecular Pathology, Institute of Pathology, University Hospital of Cologne, 50937 Cologne, Germany; johannes.braegelmann@uni-koeln.de; 6 Department of Translational Genomics, Center of Integrated Oncology Cologne-Bonn, Medical Faculty, University of Cologne, 50931 Cologne, Germany; 7 Mildred Scheel School of Oncology, Cologne, University Hospital Cologne, Medical Faculty, 50937 Cologne, Germany; 8 Pathology, Research Center Borstel, Leibniz Lung Center, Parkallee 1-40, 23845 Borstel, Germany

**Keywords:** HNSCC, CDK19, local recurrence, prognosis, recurrence-free survival

## Abstract

The Mediator complex is a central integrator of transcription and a hub for the regulation of gene expression. Cyclin dependent kinase (CDK) 19 and its paralog CDK8 are part of its kinase domain and contribute to cancer progression in different cancer entities. STAT1 is an important immune modulator and a downstream substrate of CDK8/CDK19 mediated phosphorylation. So far, little is known about CDK19’s role in head and neck squamous cell carcinoma (HNSCC) progression, its link to STAT1 activity, and related immune modulation. Immunohistochemistry for CDK19, activated pSTAT1, and PD-L1, known to be affected by STAT1, was conducted on samples of 130 primary tumors, 71 local recurrences, 32 lymph node metastases, and 25 distant metastases of HNSCC. Compared to primary tumors, CDK19 is overexpressed in local recurrences and distant metastases as well as in primary tumors that developed local recurrence after initial therapy. Patients with high-CDK19-expressing primary tumors have a significantly shorter disease-free survival. CDK19 expression correlates with pSTAT1 expression in primary tumors associated with recurrent disease, local recurrent tumors, lymph node metastases, and distant metastases. pSTAT1 expression correlates with PD-L1 expression in recurrent tumors. Our findings identify CDK19 as a potential biomarker in HNSCC to predict recurrent disease and support recent developments to target CDK19 and its paralog CDK8 in advanced cancer.

## 1. Introduction

The evolutionary and highly conserved human Mediator complex is a central integrator of transcription, serving as a hub between signaling pathways and subsequent gene expression. The Mediator consists of 33 subunits that are structurally and functionally assigned to distinct modules [1]. Aberrant activities of distinct Mediator subunits contribute to the pathogenesis of diverse disorders, such as cancer [2,3,4].

Cyclin-dependent kinase (CDK) 8 and its gene paralog CDK19 are part of the Mediator’s kinase module, which is further composed of MED12 and its paralog MED12L, MED13 and its paralog MED13L, and Cyclin C [5]. The kinase reversibly associates with the Mediator core complex and regulates the association between the Mediator and RNA polymerase II [5,6]. CDK8 and CDK19 influence gene transcription by acting as signaling pathway coactivators and corepressors, partially through phosphorylation by their enzymatic kinase function [7,8,9,10,11].

Multiple studies identified CDK8 as being involved in a broad range of cancer types including colon [12], breast [13], ovarian [14], and gastric [15] cancer, as well as acute myeloid leukemia [16]. In contrast to CDK8, less is known about the role of CDK19 in carcinogenesis. However, studies reported its involvement in colorectal [17], breast [18], ovarian [19], and prostate cancer [20,21], as well as in osteosarcoma and fibrosarcoma [8,22]. Aberrant expression of CDK8 was shown to be linked to poor prognosis in human laryngeal squamous cell carcinoma [23]. Nevertheless, the role of CDK19 in the progression and aggressiveness of head and neck squamous cell carcinoma (HNSCC) remains unclear.

Based on these observations, CDK19 emerged as a promising therapeutic target in different tumor types, leading to the development of small-molecule inhibitors impeding the activity of CDK19 and its paralog CDK8 [24,25,26,27].

Signal transducer and activator of transcription (STAT) 1 is part of the Janus kinase (JAK)/STAT signaling cascade. It regulates a wide variety of cellular processes involved in cell proliferation, cell death, and immune modulation. STAT1 is generally considered a tumor suppressor, but growing amounts of evidence suggests tumor-promoting activities [28]. Inhibition of CDK8 and CDK19 reduces STAT1 activity by repressing its phosphorylation on Ser727. Recently, STAT1 activity has been linked to PD-L1 expression, restraining antitumor immune response [11,29,30,31,32,33,34].

HNSCCs are the sixth most common cancers in humans [35,36] and the prognosis is still weak, with 30–50% of patients in UICC stages III and IV developing local or regional recurrence [36,37,38,39]. Recently, the focus of interest in many solid tumors has shifted to intratumoral immune cells. HNSCC is known to be one of the most immune-infiltrated cancer types [40]. Interactions between tumor cells and associated lymphocytes are regulated by a complex network of cells and cytokines. PD1 and PD-L1 were identified as important regulatory checkpoints whose inhibition may result in a therapeutic benefit in many solid tumors [40].

The aims of this study were to explore a potential role of CDK19 in HNSCC progression and aggressiveness and to characterize a potential implication of CDK19 on a STAT1/PD-L1 signaling axis in HNSCC, using immunohistochemistry (IHC) staining on formalin-fixed paraffin-embedded (FFPE) tissue samples of a well-characterized HNSCC cohort.

## 2. Results

### 2.1. CDK19 Is Overexpressed in Local Recurrences and Distant Metastases of HNSCC

**Figure 1:**
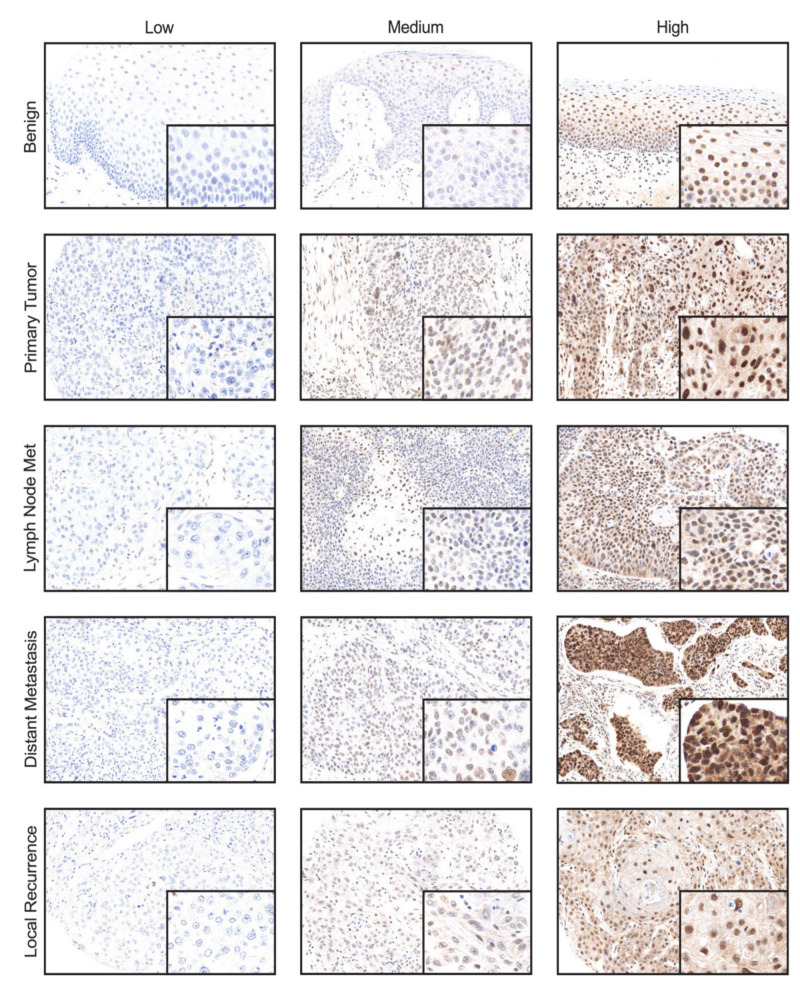
Representative IHC images showing low, medium, and high nuclear CDK19 expression in benign squamous epithelial tissue of the head and neck area, primary tumors, lymph node metastasis, distant metastasis, and local recurrence (10× magnification, insert 40×).

**Figure 2:**
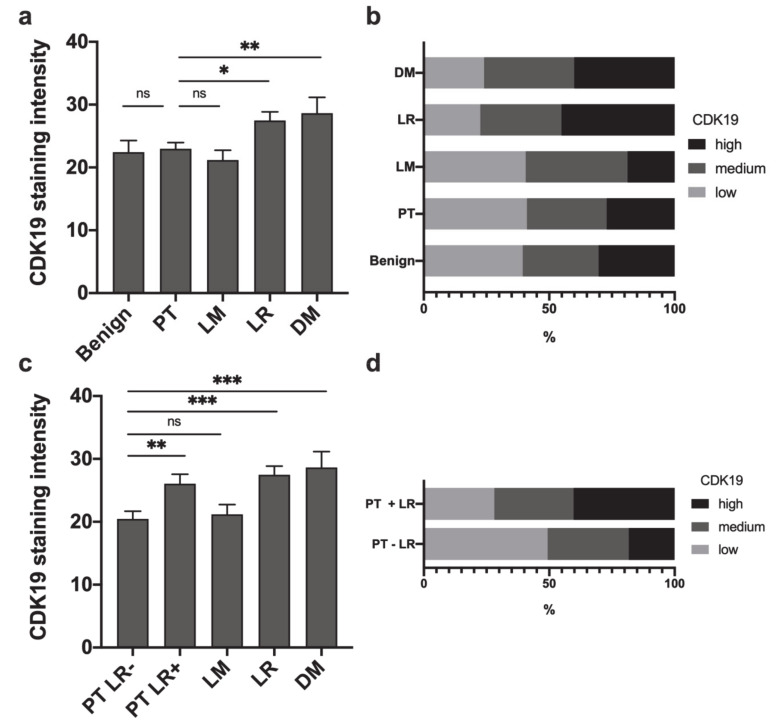
CDK19 is differentially expressed in different HNSCC tissues. (**a**,**c**) Mean nuclear CDK19 expression in different tissue types (arbitrary units). (**b**,**d**) Distribution of CDK19 expression categories in different tissues. ns = not significant; * *p* < 0.05; ** *p* < 0.01, *** *p* < 0.001.

Expression of CDK19 was examined in the tissue of primary tumors (PTs, *n* = 130), lymph node metastases (LMs, *n* = 32), distant metastases (DMs, *n* = 25), and local recurrences (LRs, *n* = 71), as well as in benign squamous epithelium from the head and neck area (benign, *n* = 33, Figure 1). IHC revealed a significantly higher expression of CDK19 in LR (mean brown 27.5, *p* = 0.008) and DM (mean brown 28.6, *p* = 0.026), compared to PT (mean brown 23.0, Figure 2a). No significant difference in expression of CDK19 between benign tissue (mean brown 22.4) and PTs, or PTs and LMs (mean brown 21.2, *p* > 0.05) was found. In a second step, CDK19 expression was grouped using the 33rd percentile into low, medium, and high expressing tumors. Only 27.1% of PTs, 45.1% of LRs, and 40% of DMs showed high CDK19 expression (Figure 2b).

### 2.2. Primary HNSCC of Patients Developing Local Recurrences Have Higher CDK19 Expression

HNSCC PTs were assessed for correlation between CDK19 expression and the presence of local recurrence. Mean CDK19 expression was significantly higher in primary tumors of patients who developed at least one LR (*n* = 71, mean brown 26.1) compared to PTs of patients who stayed recurrence-free during follow-up (*n* = 49, mean brown 20.5, *p* = 0.004). In the next step, we compared CDK19 expression of PTs with and without LR to CDK19 expression of LRs, lymph node metastases, and distant metastases. Here, we found that only PTs without subsequent local recurrence and benign tissue showed significantly lower CDK19 expression compared to LRs (*p* < 0.001) and DMs (*p* = 0.001, Figure 2c). A subset of 49.3% of PTs not resulting in LRs showed low CDK19 expression, whereas only 27.6% of PTs of patients who developed LR after primary therapy expressed low CDK19 levels (Figure 2d). CDK19 expression in PTs developing DM or LM was not significantly different from PTs not developing metastatic disease.

### 2.3. CDK19 Over-Expression in HNSCC Correlates with Reduced Disease-Free Survival

**Figure 3:**
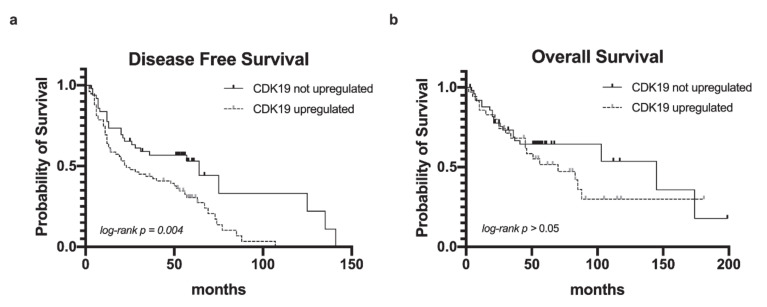
CDK19 upregulation correlates with better DFS. Kaplan–Meier diagrams showing (**a**) OS and (**b**) DFS.

To further assess the implications of CDK19 expression on patient outcome, we correlated nuclear CDK19 expression in primary tumors with disease recurrence, defined as local recurrence after primary therapy. For survival analysis, CDK19 expression-groups were dichotomized into CDK19 baseline (low) and CDK19 upregulated (medium/high). Kaplan–Meier analysis revealed CDK19-upregulated PT to be associated with a significantly shorter disease-free survival (DFS) compared to tumors lacking CDK19 expression (log-rank, *p* = 0.004, Figure 3a). The five-year DFS rates of patients who showed CDK19 upregulation in PTs compared to those without CDK19 upregulation were 30.5% and 53.0%, respectively. The significantly increased risk of recurrence in case of high CDK19 expression persisted after adjusting for p16 status using multivariate Cox analyses; this indicated that CDK19 upregulation predicts disease recurrence independently from HPV infection status (Table 1). There was a trend toward improved overall survival (OS) of patients without CDK19 upregulation, however lacking statistical significance (*p* > 0.05, Figure 3b), with a five-year OS rate of 54.8% (CDK19 upregulated in PT) and 64.4% (CDK19 not upregulated in PT). There was no significant correlation between CDK19 expression and disease-specific survival (DSS, data not shown).

### 2.4. CDK19 Over-Expression in HNSCC Correlates with Smoking Status and Alcohol Consumption, But Not with p16 Status

**Figure 4:**
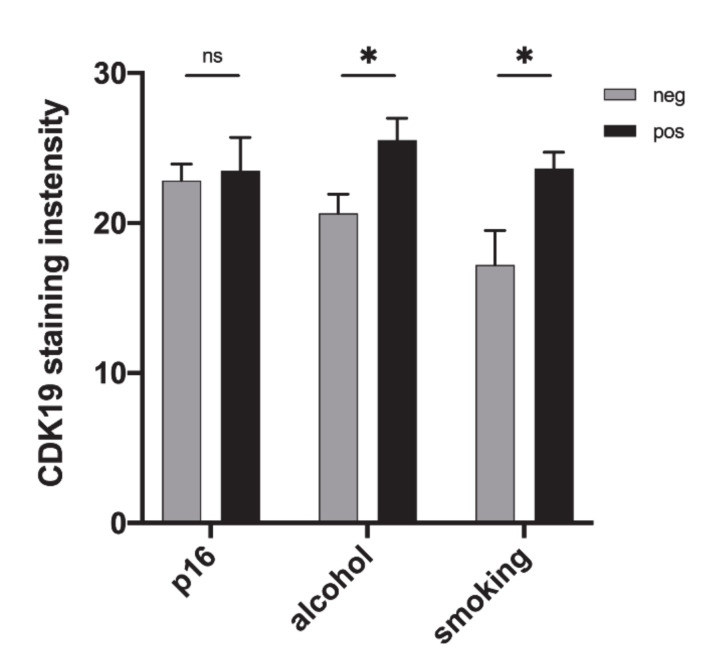
CDK19 expression correlates with clinicopathological data. Bar graphs show higher CDK19 expression in smokers and HNSCC patients with heavy alcohol consumption (arbitrary units). CDK19 expression does not correlate with p16 status. ns = not significant; * *p* < 0.05.

We found CDK19 to be expressed significantly higher in primary tumors of patients with heavy alcohol consumption (*n* = 51, mean = 25.5) compared to non-drinkers (*n* = 74, mean = 20.6, *p* = 0.014, Figure 4). The PTs of patients with a smoking history of more than one pack-year (*n* = 104, mean = 23.6) showed significantly higher CDK19 expression than tumors of non-smokers (*n* = 17, mean = 17.2, *p =* 0.026, Figure 4). However, no correlation between the number of pack-years and CDK19 expression was found.

To investigate the association of the HPV status of PT and CDK19 expression, PTs were assessed for p16 status. p16 serves as a surrogate marker for HPV infection, as recommended by the WHO and reflected in the eighth edition of the TNM classification for HNSCC [41]. No significant difference between CDK19 expression in p16-positive (*n* = 28, mean = 22.8) and p16-negative (*n* = 101, mean = 23.5, *p* = 0.789) PT was found (Figure 4). There was no association between CDK19 expression and patient age, UICC stage, or specific PT locations.

### 2.5. CDK19 Expression Correlates with pSTAT1 Expression in Primary Tumors Associated with Recurrent Disease, Local Recurrent Tumors, Lymph Node Metastases, and Distant Metastases

**Figure 5:**
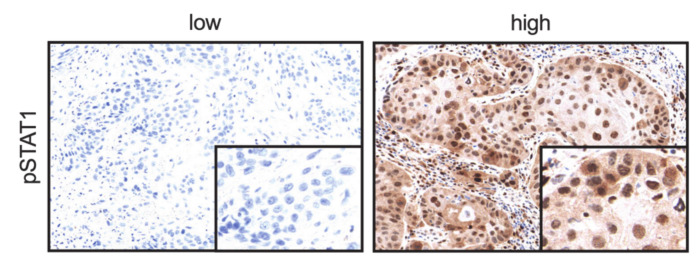
Representative images show IHC on pSTAT1 negative and high-pSTAT1-expressing HNSCC primary tumors.

**Figure 6:**
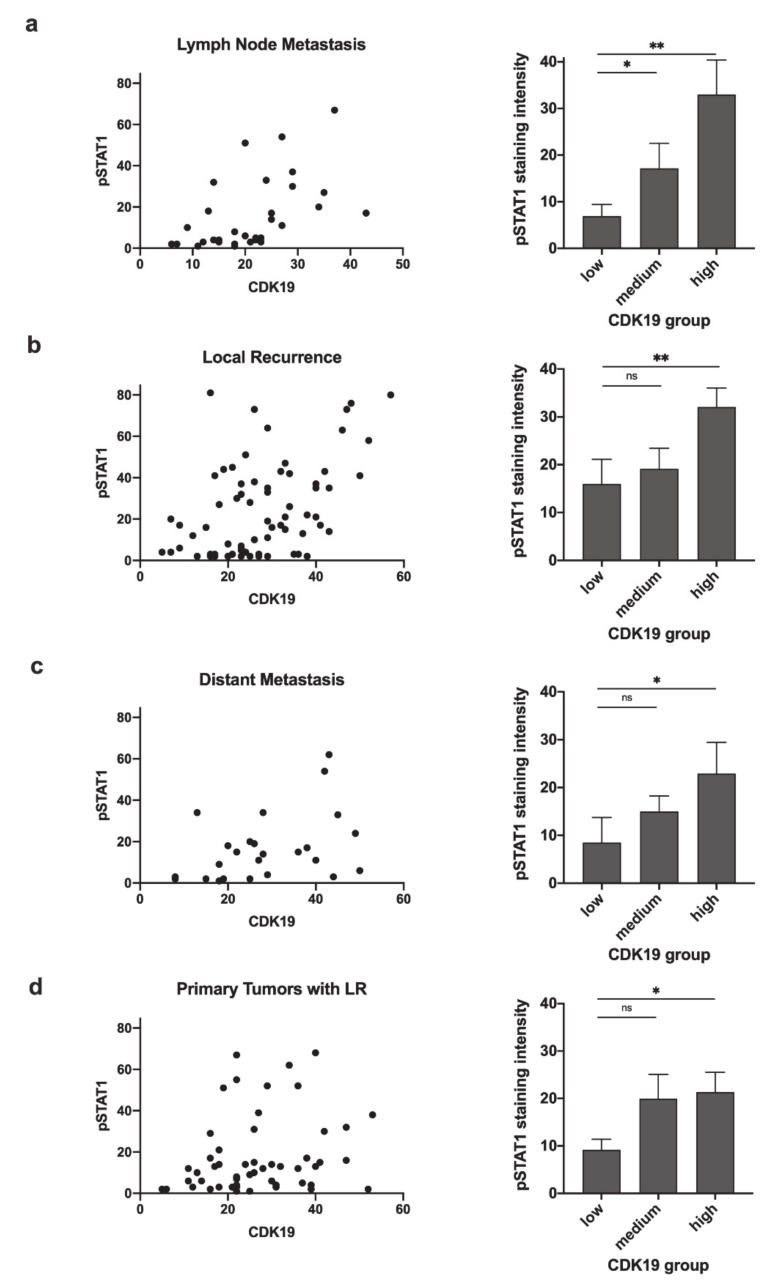
CDK19 expression correlates with pSTAT1 expression. Scatter plots and bar graphs illustrate the relationship between CDK19 expression and pSTAT1 expression in tissue of (**a**) LMs, (**b**) LRs, (**c**) DMs, and (**d**) PTs developing local recurrence after initial therapy. ns = not significant; * *p* < 0.05; ** *p* < 0.01.

HNSCCs were assessed for correlation between CDK19 expression and simultaneous expression of pSTAT1, which reflects active STAT1 signaling. Representative images showing a lack of pSTAT1 expression or nuclear pSTAT1 positivity are depicted in Figure 5. Statistical analysis revealed a strong correlation (*r*) between CDK19 and pSTAT1 expression in lymph node metastases (*r* = 0.628, *p* < 0.001, Figure 6a), a moderate correlation in local recurrences (LR) (*r* = 0.401, *p* < 0.001, Figure 6b), and distant metastases (*r* = 0.430, *p* < 0.05, Figure 6c), but no significant correlation within primary tumors (PTs) or benign tissue. In the next step, PTs were regrouped into PTs of patients who developed LR after therapy and PTs not associated with LR. Here, we found a moderate but significant correlation between CDK19 and pSTAT1 expression in the first group (*r* = 0.300, *p* < 0.05, Figure 6d), but no correlation in the second group.

### 2.6. pSTAT1 Expression Correlates with PD-L1 Expression in Local Recurrences

**Figure 7:**
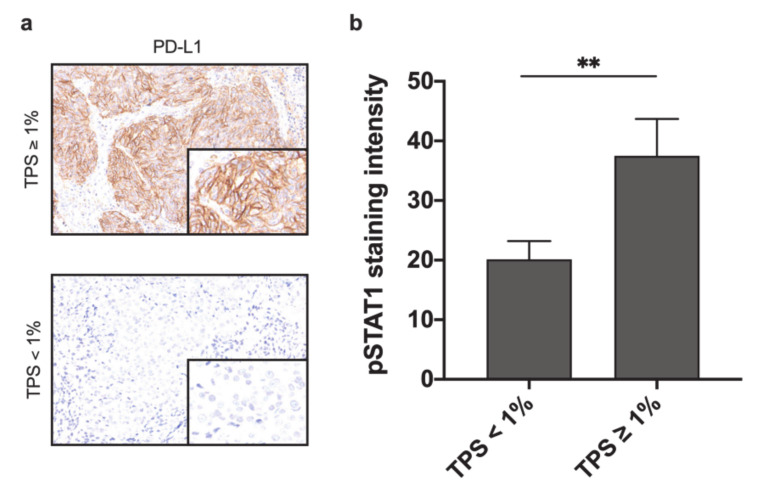
pSTAT1 expression is associated with PD-L1 expression in HNSCC local recurrence. (**a**) Representative images of tumors with TPS ≥ 1% and TPS < 1%. (**b**) Difference in mean pSTAT1 expression in PD-L1 positive (TPS ≥ 1%) and negative (TPS < 1%) tumors. ** *p* < 0.01.

To obtain further evidence for a possible role of CDK19-mediated STAT1 phosphorylation and its effect on tumor immune microenvironment in HNSCC, CDK19, and pSTAT1 expression were correlated with PD-L1 status. For PD-L1 expression, the tumor positivity score (TPS) was assigned, resulting in 45 PD-L1-negative LRs (TPS < 1%), and 20 PD-L1-positive LRs (TPS ≥ 1%, Figure 7a). Comparing the pSTAT1 expression in these groups, we observed significantly higher pSTAT1 expression in PD-L1 positive LRs (mean brown 20.1) compared to the group of PD-L1 negative LRs (mean brown 37.5, *p* = 0.006, Figure 7b). No correlation between PD-L1 and pSTAT1 expression was found in PTs, DMs, and LMs. There was no correlation between CDK19 expression and PD-L1 status.

## 3. Discussion

CDK19 and its paralog CDK8 are known to promote different cancer-related pathways in several solid tumors, such as TGF-β/BMP-induced epithelial–mesenchymal-transition (EMT), and NFκB-mediated gene transcription [7,8,10,11]. Recently, CDK19 and CDK8 emerged as promising potential therapeutic targets in cancer therapy [24,25,26,27].

To the best of our knowledge, this is the first study to investigate the potential role of CDK19 in the progression and aggressiveness of HNSCC. Our data showed an over-expression of CDK19 in primary tumors of patients that developed local recurrence after primary therapy, and the prognostic value of CDK19. Given the high rate of local recurrence and controversial discussion on the use of radiotherapy and chemotherapy in patients diagnosed with HNSCC [39,42,43,44] to prevent recurrent disease, CDK19 might serve as a valuable biomarker to identify patients who benefit from aggressive therapies, once validated on independent patient cohorts.

Notably, survival analysis revealed a significantly longer period of DFS in HNSCC primary tumors lacking CDK19 expression compared to CDK19-positive tumors with a difference in median DFS of 3.4 years. Surprisingly, our data showed no significant effect of CDK19 expression on OS and DSS, despite the significantly higher rate of local recurrence in patients with high- CDK19-expressing tumors. However, this result might be caused by a small sample size in our cohort, as there was a clear trend toward better OS in the CDK19-negative group compared to CDK19-positive tumors. Additionally, it is known that, in many cases, HNSCC patients suffer from lifestyle-associated cardiovascular comorbidities and, therefore, carry a high risk of non-tumor-associated premature death [45].

Besides conventional prognostic factors such as TNM stage and grading, positive p16 status, that is a surrogate for HPV infection, is the only routinely-used biomarker for better survival and fewer cases of local recurrence in oropharyngeal HNSCC [46]. Our data showed no connection between p16 status and CDK19 expression in HNSCC, indicating CDK19 to be an HPV-independent marker for recurrent disease. We assume that CDK19 evaluation on biopsies of HNSCC, which were taken at the time of initial diagnosis, will show similar results. Thus, CDK19 might support clinical management to risk-stratify patients before treatment. Further analyses are needed to investigate whether CDK19 might predict response to radiotherapy, which is established as first-line therapy for HNSCC next to surgery. Furthermore, CDK19 expression was significantly higher in HNSCC patients with a history of smoking or heavy alcohol consumption, implying a potential influence of these agents on CDK19 expression in HNSCC.

Previous functional analysis revealed CDK8/CDK19 to phosphorylate STAT1 on Ser727 with subsequent STAT1 activation [11]. STAT1 is known to be immunomodulatory and is associated with PD-L1 [28,34], indicating a potential effect of CDK19 on tumor immune microenvironment. Thus, to further explore the signaling axis between CDK19, pSTAT1 (Ser727), and PD-L1, we correlated the expression of these proteins in different types of HNSCC tissue, assuming that high expression reflects active signaling. These analyses revealed a significant correlation between CDK19 and pSTAT1 expression in all investigated types of HNSCC tissue, except for benign tissue and primary tumors without associated local recurrence. This result might indicate that CDK19 activity promotes STAT1 activity only in aggressive tumors and metastases; however, functional in vitro analysis would be needed to validate our tissue findings further. Furthermore, we found pSTAT1 to be significantly higher expressed in PD-L1-positive recurrent tumors; however, no correlation was found in primary tumors or metastases. In addition, there was no correlation between CDK19 and PD-L1 expression indicating a potential link between CDK19-STAT1-PD-L1 in a distinct subset of aggressive HNSCC. The complex regulatory network of PD-L1 expression and activity, as well as considering the multiple functions of both STAT1 and CDK19, might explain the lack of significance in the direct correlation between CDK19 and PD-L1, and the presence of CDK19 expressing tumors without STAT1 activation.

Our study revealed CDK19 as a potential biomarker in HNSCC primary tumors to predict local recurrence and disease outcomes after the initial therapy, and therefore to help risk-stratify patients. The differences between low, medium, and high CDK19 IHC staining were differentiable in the vast majority of samples and could be evaluated by pathologists in daily routine. Consistent with previous data in other tumor types, our study indicated that CDK19 might contribute to HNSCC progression and aggressiveness, supporting recent developments to target CDK19 and its paralog CDK8 in advanced cancer.

## 4. Materials and Methods

### 4.1. Patient Data and Tumor Material

This study was conducted following the Declaration of Helsinki. The protocol was approved by the Ethics Committee of the University of Lübeck (project code AZ 16-277; approval date 18th November 2016).

The cohort comprised tissue from 163 patients with HNSCC (21.4% oral, 34.6% oropharyngeal, 12.6% hypopharyngeal, and 31.4% laryngeal; 77% were male patients and 23% female; 76.5% were p16-negative and 23.5% p16-positive; 47.2% were UICC stage I/II and 52.8% UICC stage III/IV; 54.6% had at least one LM, and 43.4% had no regional LMs; 22.7% developed DM and 77.3% did not; 52.1% developed LR and 47.9% did not). Sample numbers with available tissue and successful IHC, which could be considered for analysis, were 130 primary tumor tissues, 32 lymph node metastases, 25 distant metastases, 71 recurrent tumor tissues, and 33 samples of normal squamous epithelium from the head and neck area (benign).

Patients were diagnosed between 2012 and 2015 in the Institute of Pathology of the University Medical Center Schleswig-Holstein, Campus Lübeck, and treated in the Department of Otorhinolaryngology. Survival data, patient characteristics, and social histories were obtained from medical records.

Using representative FFPE tumor tissues, tissue microarrays (TMAs) were created[47].

### 4.2. Immunohistochemistry

IHC staining was performed using the Ventana Discovery System (Ventana Medical System, Oro Valley, AZ, USA) [48]. Briefly, slides were incubated at room temperature with primary antibodies: anti-CDK19 polyclonal rabbit (HPA007053, Sigma, St. Louis, MO, USA), pSTAT1 (D4X3C, Cell Signaling, Danvers, MA, USA), PD-L1 (E1L3N, Cell Signaling, Danvers, MA, USA), and detected with the ultraView Universal DAB Detection Kit (Ventana Medical System, Tucson, AZ, USA).

### 4.3. Evaluation of Immunohistochemical Staining

CDK19 and pSTAT1 stained slides were scanned and digitalized using a Ventana iScan HT scanner (Ventana, Tucson, AZ, USA). As described before [47,49]; the semi-automated image analysis software Tissue Studio (Definiens Developer XD 2.0, Definiens Inc., Carlsbad, CA, USA) was applied to quantify digital data. Briefly, tumor cell areas were manually annotated for each TMA core as Region of Interest ROI by a pathologist, and by using arbitrary units, a continuous spectrum of tumor cell nuclei brown staining intensity was obtained. For statistical evaluation, we analyzed three tissue core samples of the same patient and calculated the arithmetic mean for the respective triplets.

Two independent pathologists evaluated PD-L1 stained slides. The estimated percentage of tumor cells that showed partial or complete membrane PD-L1 staining (tumor positivity score) is reported separately for each core. Three tissue core samples of the same patient were analyzed, and the arithmetic mean of the respective triplet was calculated. Results were regrouped into therapeutically relevant categories (TPS < 1% and TPS ≥ 1%).

### 4.4. Statistical Analysis

Statistical analyses were performed using IBM SPSS Statistics for Windows (Version 26.0, IBM Corp., Armonk, NY, USA) and Prism 8 (GraphPad Software, LCC, San Diego, CA, USA). An unpaired two-tailed *t-*test was applied to compare CDK19 expression between p16-positive and p16-negative PTs, PTs of smokers and non-smokers, PTs of patients with and without a history of heavy alcohol consumption, PTs with or without metastatic disease, PTs developing or not developing LR, PD-L1 positive (TPS ≥ 1%) and negative (TPS < 1%) tissues, and between PTs, LMs, DMs, and LRs. Univariate analysis of variance (ANOVA) was applied to discriminate CDK19 expression in different UICC stages and different PT locations. Spearman’s rho was used to investigate the correlation between CDK19 expression and pSTAT1 expression in different tissue types, as well as the potential correlation between the number of pack-years and CDK19 expression PTs of smokers. OS, DSS, and DFS were calculated using the Kaplan–Meier method and log-rank test for statistical significance. Multivariate Cox regression was used for adjustment to p16-status. *p*-values less than 0.05 were considered statistically significant. Bar graphs reveal mean as columns and standard error of the mean (SEM) as error bars.

## Figures and Tables

**Table 1:** Hazard ratio (HR), 95% confidence interval (CI)l, *p*-value of CDK19, and p16 status in multivariate Cox analysis.

Status	HR	95% CI	*p-*Value
CDK19 upregulated	2.058	1.274–3.325	0.003
p16 positive	0.485	0.276–0.853	0.012

## Abbreviations

CDK	Cyclin-dependent kinase
DFS	Disease-free survival
DM	Distant metastasis
DSS	Disease-specific survival
FFPE	Formalin-fixed paraffin-embedded
HNSCC	Head and neck squamous cell carcinoma
IHC	Immunohistochemistry
JAK	Janus Kinase
LM	Lymph node metastasis
LR	Local recurrence
OS	Overall survival
PD-L1	Programmed-Death Ligand 1
PT	Primary tumor
ROI	Region of Interest
STAT1	Signal Transducer and Activator of Transcription 1
TMA	Tissue microarray
TPS	Tumor positivity score
